# Pilot study of hemoglobinopathies in newborns of the Rafael Calvo maternity clinic of Cartagena, Colombia

**Published:** 2012-09-30

**Authors:** Ciro Cesar Alvear, Miriam Barboza, Maricela Viola, Carlos Moneriz, Luz Marina Araque

**Affiliations:** a Facultad de Medicina, Universidad de Cartagena. E-mail: mirbar2001@yahoo.com; b Clinical Biochemistry. E-mail: cicealse@yahoo.es

**Keywords:** Hemoglobinopathies, newborn, isoelectric focusing electrophoresis, Incidence, epidemiology, anemia

## Abstract

**Introduction:**

The hemoglobinopathies are a heterogeneous group of congenital anemias from Africa, Asia and the Mediterranean. Due to the migration of this population have spread worldwide, especially in Latin America and the Caribbean region, which Cartagena de Indias is included, with a large proportion of people of African descent. The lack of routine programs that include an appropriate methodology for precise identification of those affected and carriers, impossible to know the real behavior of this disease in our country and an early and appropriate to the patients before the disease manifests itself and produce its serious consequences.

**Objective:**

To estimate the incidence and describe the epidemiological profile of hemoglobinopathies in newborns Rafael Calvo Maternity Clinic of Cartagena, in the period from January to June 2010.

**Methods:**

Prospective descriptive study of a population of 1,729 newborns. Samples were collected cord blood on filter paper. Isoelectric focusing electrophoresis (IEF )was used to separate the haemoglobins.

**Results:**

94.4% (1,633 samples) were normal (hemoglobin FA), 4.5% (78 samples) were heterozygous for haemoglobin S (HbFAS), 1% (17samples) were heterozygous for haemoglobin C (hemoglobin FAC) and 0.1% (1 sample) was double heterozygous SC (hemoglobin FSC).

**Conclusion:**

Due to the high incidence of hemoglobinopathies found in this pilot study highlights the importance and necessity of establishing an obligatory neonatal screening in the city of Cartagena, in order to make a timely diagnosis and monitoring of affected and carrier.

## Abstract (es)

**Introducción:**

Las hemoglobinopatías comprenden un grupo heterogéneo de anemias congénitas originarias de África, Asia y la cuenca mediterránea. Debido a las migraciones de este grupo poblacional se han esparcido en todo el mundo, especialmente en América Latina y la región Caribe, en la cual está incluida Cartagena de Indias, con una gran proporción de población afrodescendiente. La inexistencia de programas rutinarios para la identificación precisa de los afectados y portadores, imposibilita conocer el comportamiento real de esta enfermedad en nuestro medio, el manejo temprano y adecuado a los pacientes, antes que la enfermedad se manifieste y produzca sus graves secuelas.

**Objetivo:**

Estimar la incidencia y describir el perfil epidemiológico de las hemoglobinopatías, en recién nacidos de la Clínica Maternidad Rafael Calvo de Cartagena, en el período de enero a junio del año 2010.

**Método:**

Estudio descriptivo prospectivo. Se tomó muestra de cordón umbilical recolectada en papel de filtro, a una población de 1,729 recién nacidos, empleando la técnica de electroforesis de punto isoeléctrico.

**Resultados:**

El 94.4 % (1,633 muestras) fueron normales (hemoglobina FA), el 4.5% (78 muestras) fueron heterocigotos para hemoglobina S (hemoglobina FAS), el 1% (17 muestras) fueron heterocigotos para hemoglobina C (hemoglobina FAC) y el 0.1% (1 muestra) fue doble heterocigoto SC (hemoglobina FSC).

**Conclusión:**

debido a la alta incidencia de hemoglobinopatías encontrada en este estudio piloto, se resalta la importancia de establecer un programa de tamizaje neonatal en la población de Cartagena, a fin de realizar un oportuno diagnóstico y seguimiento de los afectados y portadores.

## Introduction

The hemoglobinopathies are a heterogeneous group of inherited disorders including: hemoglobin variants, thalassemia and hereditary persistence of fetalhemoglobin. Hemoglobinopathies are the most common inherited diseases in humans affecting approximately 5% of world population^1^. The abnormal genes of hemoglobin originated from Africa, Asia and the Mediterranean, and due to the migration of this population have spread worldwide, especially in Latin America. The Colombian Caribbean is located in this geographical area and has a high prevalence of sickle-cell anemia or drepanocytosis, especially in those countries with a high proportion of African people (Brazil, Guyana, Panama and Caribbean islands)^2^ ^,^ ^3^. The alteration in the structure and function of hemoglobin causes deficiencies in the transport of oxygen by red blood cells, resulting in tissue damage that is reflected in total or partial incapacity, and in many cases death^4^.Sickle cell trait is very common in those regions where African slaves arrived, reaching prevalence's of 8% and has been reported in Brazil, with mixed racial populations, the gene has a prevalence of 5-6%^5^.

Morbidity, mortality and quality of life of patients depend on the values of HbF in the early stages of life, but in later stages will be linked to earlier diagnosis, socio-sanitary conditions and medical care^6^ ^-^ ^11^. Each year about 120,000 children are born with sickle cell anemia in sub-Saharan Africa^12^ and less than 2% survive the first years of life. In the U.S. the median survival exceeds 50 years^13^, demonstrating that the level of specialized care is directly related to the progress and outcome of disease.

In Colombia there are no national systematic studies on the epidemiology of hemoglobinopathies. However, partial data are taken in some populations at risk in the departments of Chocó, Antioquia and Valle del Cauca and can be extrapolated to understand the magnitude of the problem. In a study of a black population in Salahonda (Tumaco-Colombia)were found in 10% of sickle cell trait and 1% higher hemoglobinopathies^14^. In other studies were found frequencies of hemoglobinopathies in the range from 3.8% to 10% using electrophoresis techniques^15^ ^-^ ^17^. A neonatal screening performed in the city of Cali (Colombia) were found 2.4% of sickle cell trait (AS), 1.4% of heterozygotes for hemoglobin C (HbAC), 0.02% heterozygous for hemoglobin D (HbAD ), 2 homozygous for hemoglobin C (CC) and no reported cases of homozygous hemoglobin S (SS)^18^. Finally, a study in the city of Buenaventura (Colombian Pacific) with a population of 92% Afro-Colombian, was found in 399 samples from the umbilical cord, that 5.8% were heterozygous for hemoglobin C (FAC), 4.8% were heterozygous for hemoglobin S (FAS), 0.5% were heterozygous for hemoglobin G (FAG), 1 was heterozygous for hemoglobin D (FAD) and 1 was heterozygous S and C combined (FSC)^19^.

Moreover, sickle cell anemia is not included in the Obligatory Health Plan (POS) in Colombia,which complicates their specialized care, given the many features and clinical manifestations affecting the patient's life from birth and throughout his life.

The objective of this study was to estimate the incidence and describe the epidemiological profile of hemoglobinopathies in newborns Rafael Calvo Maternity Clinic of Cartagena, although no previous studies of systemic, is known for its characteristics of high prevalence of disease.

##  Materials and Methods

A prospective, descriptive study of 1,729 infants born during the period from January to June 2010 at ESE Rafael Calvo Maternity Clinic of Cartagena was conducted to investigate the epidemiological profile of hemoglobinopathies.In this second-level hospital born around 65% of the population of Cartagena and its surroundings, belonging to the socioeconomic level 1, 2 and 3.To estimate the sample size, we took into account a prevalence of 5%^1^, a confidence interval of 95% and a margin of error of 1.25% for a minimum number of samples 1,168. Samples were collected cord blood on filter paper.

### Exclusion criteria:

Neonates less than 36 weeks (confirmed by obstetric ultrasound supplied by the mother, clinical examination and date of last menstrual period), those weighing less than 500 g and unsatisfactory samples (diluted).

### Analytical method:

For sample processing, was used the technique of the isoelectric focusing electrophoresis (IEF), in which the separation of hemoglobin was performed by loading a blood sample on an agarose gel containing ampholytes with a pH between 6 and 8 and quantified by densitometry. Was used a pool of blood as a quality control provided by ROPSOHN THERAPEUTICS LTDA, containing hemoglobin F, A, S and C. The IEF technique has a high sensitivity and precision, allowing analyzing a large number of samples,achieving to detect all hemoglobinopathiesof clinical significance. For statistical analysis of data was used EPI-INFO software version 3.5.1 and Excel-2007. Nonparametric variables were analyzed with the Mann-Whitney test.

### Ethical considerations: 

This research was approved by the ethics committee of the University of Cartagena. 

## Results

**Figure 1:**
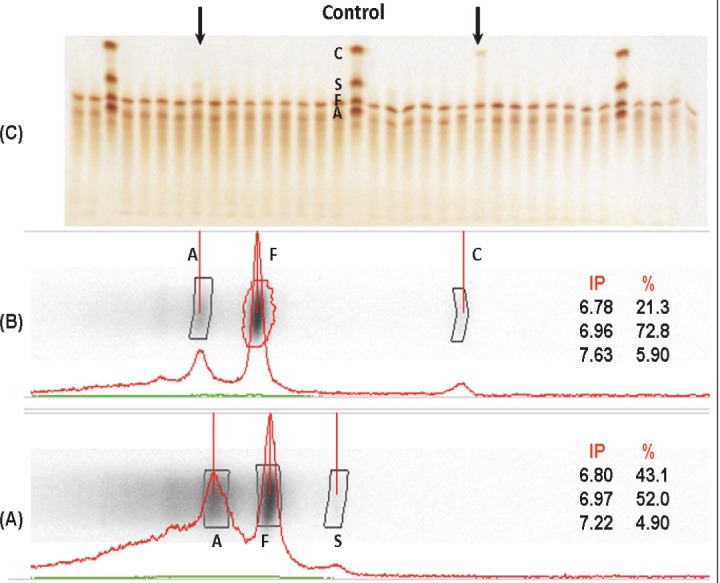
Densitometric analysis of two positive cases, one for Hb AS (A) and one for Hb AC (B). Samples run by isoelectric focusing electrophoresis (C) in agarose gel, using hemoglobin control AFSC.Arrows indicate positive cases. IP = isoelectric point, % = Percentage of hemoglobin type.

**Figure 2:**
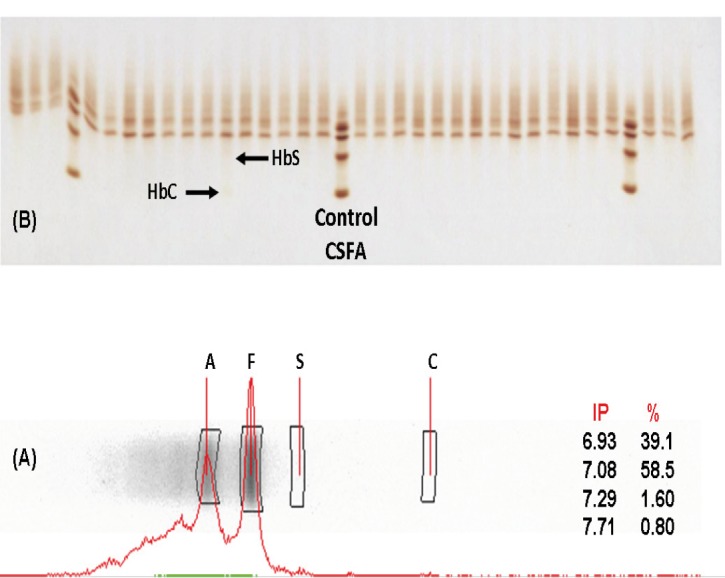
(A) Densitometric analysis of the positive case for hemoglobin ASC. (B) Samples run by isoelectric focusing electrophoresis in agarose gel. Arrows indicate positive case.

A total of 1729 samples obtained from umbilical cord were analyzed, of which 44.7% (773) were females, 51.2% (886) were males and 4% (70) no data record of the gender. 94.4% of the samples showed a normal pattern of hemoglobin (HbFA), 4.5% corresponded to the sickle cell trait (HbFAS), 1% of the samples showed the trait for hemoglobin C (HbFAC) and 0.1% were double heterozygous SC (HbFSC), for an incidence of 5.6% hemoglobinopathies. Figure 1 shows densitometric analysis of two positive cases, one for Hb AS and one for Hb AC; while in Figure 2 shows densitometric analysis of the positive case for hemoglobin ASC.

Table 1 shows the general distribution by gender, origin, maternal age, weight and size of the newborn using the median and interquartile range according to criteria of normality. It is pertinent to note that in 6 of the positive cases the data was not recorded sex (four variant corresponded to the AS and two at the AC).

**Table 1:**
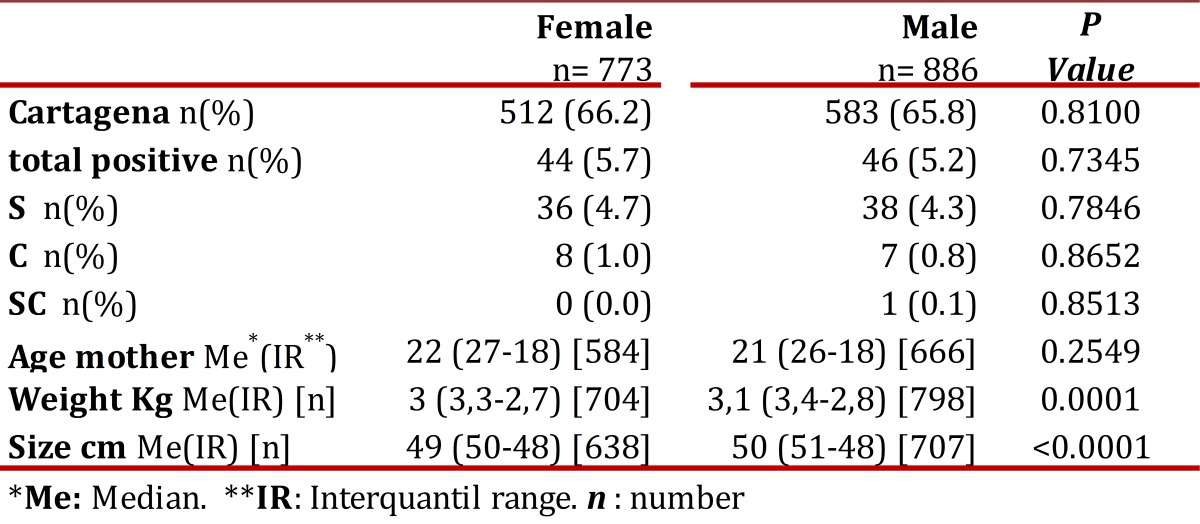
General distribution by gender, origin, maternal age, weight and size of the newborn using the median and interquartile range according to criteria of normality.

## Discussion

We demonstrated in this research, a 5.6% incidence of hemoglobinopathies in newborns from the main maternity hospital in Cartagena, being the most common variant sickle cell trait with 81% of all positive cases (78/96). Due to the high incidence and prevalence of hemoglobinopathies in Colombia it is necessary that these genetic disorders are of priority in public health actions, if we follow the WHO criteria^20^.

We observed in this study, the incidence of hemoglobinopathies is consistent with that reported worldwide and with findings in other regions of Colombia. In studies in Colombia, the most common hemoglobinopathies corresponds to the sickle cell trait, except that carried out in Buenaventura predominated for hemoglobin C trait and followed in importance by sickle cell trait with a similar percentage of cases found in Cartagena.

Among these disorders, the most important are those related to alterations in the beta chain of hemoglobin, where the sickle cell anemia (FA) is the most severe and corresponds to its homozygous form (SS). It can also occur as heterozygous (AS), producing the so-called sickle cell trait.

People with sickle cell trait (HbAS) do not usually have clinical symptomsbut can transmit the disease to their children. It is possible that a person with sickle cell trait manifest complications of sickle cell anemia, as splenic sequestration, painful crisis and, most unusually, sudden death. This can occur under extreme conditions of high altitude (during an airplane flight), increased pressure (for diving), low oxygen (strenuous physical exercise or training for athletic competition), and in cases of dehydration^21^ ^-^ ^23^.

Therefore, the screening of newborns in Cartagena de Indias, may allow early diagnosis and timely treatment and other preventive measures such as adequate genetic counseling and education. Currently, 40 states of the American Unionconduct tests to determine whether a newborn is a carrier of genes for sickle cell anemia. If the carrier has children with another person who is a carrier, this couple has a 25% chance that your child have sickle cell disease.However, improvements in treatment (often with penicillin, folic acid, analgesic, antipyretic and blood transfusions), advances in prevention efforts and progress in the education of parents of children with sickle cell disease, have led to significantly reduce infant mortality from this disease in the United States^24^.

Determining the actual incidence rates of hemoglobinopathies can be achieved by a pilot study in countries like Colombia, where not done this type of screening and which have made in Cartagena. This city has shown that phenotypic variants AS and AC are very common. These results should alert health authorities, as hemoglobinopathies constitutes a public health problem in Colombia, it is suggested to implement a massive program of neonatal screening for hemoglobinopathies in high-incidence regions such as Cartagena, would improve the cost-ffectiveeness of screening, so it should be integrated into the primary health system in the country./p> 

## References

[1] WHO (2006). Consejo ejecutivo Acta 118.

[2] Doménech E, Castro J, Barroso F, Sanjurjo P, Baldellou A (2001). Diagnóstico y tratamiento de las enfermedades metabólicas hereditarias..

[3] Saenz Renauld GF (1988). Hemoglobinopathies in Caribbean Basin countries.. Rev Biol Trop.

[4] Fosdal MB, Wojner-Alexandrov AW (2007). Events of hospitalization among children with sickle cell disease.. J Pediatr Nurs.

[5] Salomón-Cruz J, Gómez-Valencia L, Morales-Hernández A (2006). Estudio clínico y genético de tres casos de anemia de célulasfalciformes.. Sal Tab.

[6] Smith LA, Oyeku SO, Homer C, Zuckerman B (2006). Sickle cell disease: a question of equity and quality.. Pediatrics.

[7] De montalembert M (2012). Prise en charge des enfants drépanocytaires : un travail d'équipe..

[8] pediatrics Aao (2002). Health supervision for children with sickle cell disease.. Pediatrics.

[9] Davies SC, Cronin E, Gill M, Greengross P, Hickman M, Normand C (2000). Screening for sickle cell disease and thalassaemiaa systematic review with supplementary research.. Health Technol Assess.

[10] Cervera  BA, Cela  JME (2007). Anemia FalciformeManejo en Atención Primaria. Rev Pediatr Aten Primaria.

[11] Henthorn JS, Almeida AM, Davies SC (2004). Neonatal screening for sickle cell disorders.. Br J Haematol.

[12] Rahimy MC, Gangbo A, Ahouignan G, Adjou R, Deguenon C, Goussanou S (2003). Effect of a comprehensive clinical care program on disease course in severely ill children with sickle cell anemia in a sub-Saharan African setting.. Blood.

[13] Steinberg MH (1999). Management of sickle cell disease.. N Engl J Med.

[14] Muñoz N, Pereira F, Sáenz I (1994). Hemoglobinas anormales en Salahonda (Tumaco).. Acta Pediatr Colomb.

[15] Pereira FD, Sáenz I, Pereira FD, Sáenz I (2009). Hemoglobinopatías en niños.. Colomb Med.

[16] Bernal MdP, Giraldo A, Bermúdez AJ, Moreno E, Bernal MdP, Giraldo A (1995). Estudio de la frecuencia de hemoglobinopatías en las islas de San Andrés y Providencia, ColombiaStudy of the frequency of hemoglobinopathies in the Colombian Caribbean Islands. Biomédica.

[17] Silva SJR, Malambo D, Silva DF, Fals E, Fals O, Silva JR (1998). Tamizaje de hemoglobinopatías en muestra de la población infantil de Cartagena.. Pediatría.

[18] Satizabal J, Neuta P, Muñoz J, Somoyar P (2004). Incidencia de hemoglobinopatías en neonatos de Cali.. Salud Uninorte.

[19] De Bernal M, Collazos A, Bonilla RD, Tascón EP, Bernal MD, Collazos A (2010). Determination of the prevalence of hemoglobin S, C, D, and G in neonates from Buenaventura, Colombia.. Colomb. Med.

[20] Penchaszadeh VB, Christianson AL, Giugliani R, Boulyjenkov V, Katz M (1999). Services for the prevention and management of genetic disorders and birth defects in developing countries.. Community Genet.

[21] Tapia-Gonzalez JL, Sánchez A, Uzcátegui E, Guzmán J, Camarata F (2009). InfartoEsplenicoAnemiaFalciforme. Rev. Venez. Cir.

[22] Ruiz Semba E, Garavito Renteria J, Jimenez Bustamante J, Arteaga Caro R, Garcia Del Aguila JL, Chavez Gil V (2006). Acute abdominal pain due to splenic infarction in a patient with heterozygous sickle cell disease exposed to high altitude.. Rev Gastroenterol.

[23] L. N. (2009). Health Rare athlete deaths spur sickle cell trait testing Seattle Times Newspaper.

[24] Lees CM, Davies S, Dezateux C (2000). Neonatal screening for sickle cell disease.. Cochrane Database Syst Rev.

